# Ecological Integrity Impairment and Habitat Fragmentation for Neotropical Macroinvertebrate Communities in an Agricultural Stream

**DOI:** 10.3390/toxics10070346

**Published:** 2022-06-22

**Authors:** Silvia Echeverría-Sáenz, Rocío Ugalde-Salazar, Meyer Guevara-Mora, Francisco Quesada-Alvarado, Clemens Ruepert

**Affiliations:** 1 Central American Institute for Studies on Toxic Substances (IRET), Universidad Nacional, Heredia 40101, Costa Rica; maria.ugalde.salazar@una.ac.cr (R.U.-S.); francisco.quesada.alvarado@una.ac.cr (F.Q.-A.); clemens.ruepert@una.ac.cr (C.R.); 2 Doctorado en Ciencias Naturales para el Desarrollo (DOCINADE), Instituto Tecnológico de Costa Rica, Universidad Nacional, Universidad Estatal a Distancia, Heredia 40101, Costa Rica; 3 Laboratorio de Entomología (LEUNA), Escuela de Ciencias Biológicas, Universidad Nacional, Heredia 40101, Costa Rica; meyer.guevara.mora@una.ac.cr

**Keywords:** pesticides, Volcán River, Costa Rica, nitrates, community ecotoxicology, river habitat fragmentation

## Abstract

The Volcán River watershed in the south Pacific of Costa Rica comprises forests, small urban settlements, cattle fields, and intensive agriculture (mostly pineapple and sugarcane). The ecological integrity and quality of its waters was assessed from 2011–2013 and 2018–2019 by means of physical–chemical parameters (pH, conductivity, temperature, DO, DBO, nitrate, total phosphorus, and pesticide residues) and benthic macroinvertebrate (MI) sampling in eight sites (Volcán, Cañas, and Ángel Rivers, and Peje and Maura streams), resulting in high ecological integrity in all sites except the Peje stream, which is polluted with nitrates and pesticides. Only in this stream was there a marked seasonal variation in the abundance of 16 MI families including Leptohyphidae, Leptophlebiidae, Philopotamidae, Glossossomatidae, and Corydalidae, among others, whose presence was limited exclusively to the dry season (December to April), disappearing from the stream in the rainy season, with corresponding peaks in nitrate (max 20.3 mg/L) and pesticides (mainly herbicides and organophosphate insecticides). The characteristics of the watershed, with large areas of forest and excellent water quality, allow for the re-colonization of organisms into the Peje stream; however, those organisms are incapable of development and growth, providing evidence of a contaminant-driven habitat fragmentation in this stream during the rainy season.

## 1. Introduction

The ecological integrity of a river or stream, meaning its suitability to offer optimal conditions for the establishment of biotic communities, is determined by a series of environmental factors [1]. Amongst the most relevant are those related to hydrology (e.g., water flow, current velocity, seasonality, and frequency of floods); habitat characteristics (quality and quantity of the riparian vegetation, substrate, river channel width, depth, and morphology); chemical and physical variables (alkalinity, temperature, dissolved oxygen, pH, turbidity, and xenobiotic presence); energy sources (nutrients, solar radiation, primary production, and organic matter); and also biotic factors related to food availability, intra and inter-species competition, reproduction rates, or predation [2].

In case of anthropogenic or naturally originated alterations of any of these factors, the availability of necessary resources for aquatic life, or the fulfillment of the ecological roles of each species, can be limited [2]. Therefore, any modification within a watershed can potentially reflect temporal and spatial variations in the ecological integrity of a river. Agricultural watersheds, for example, pose several challenges related to land use changes [3], deforestation of riparian corridors [4,5], erosion, sedimentation, changes in channel morphology [6,7], and use of fertilizers and pesticides which exert pressure on the receiving superficial waters and their biota [8,9]. Freshwater macroinvertebrate (MI) communities in continental waters worldwide have been severely affected by those pressures, with consequent threats on taxa richness and biodiversity [10,11,12].

The Neotropical region has the particularity of maintaining optimal temperature conditions for crops throughout the year [13]; therefore, agriculturally related stress factors are permanent, with no resting or recovery periods for the streams. Multiple authors have provided evidence of high risk for invertebrates and primary producers derived from pesticides detected in tropical agricultural watersheds [14,15,16,17,18,19,20]. Moreover, these stressors coexist with other Neotropical conditions such as high variation in rainfall due to climate change (especially in Central America), which might affect seasonal patterns of biota or even produce mortality in heavy drought or flood events [21].

The capability of aquatic biota to colonize or migrate through a specific river stretch can be inhibited by many different factors such as high suspended sediment loads, frequent floods or extreme drought events, high xenobiotic concentrations, and low input of allochthonous material because of riparian forest absence, among others [22,23,24]. Therefore, the longitudinal connectivity of a watershed (from the lower to the upper parts of the basin) can be compromised or interrupted where agriculturally related stress factors take place, creating a fragmentation of the aquatic habitat, similar to what can be found in a dammed site, but produced by a pollution barrier [25].

Studies around the globe have evidenced a profound effect of river networks habitat fragmentation (especially produced by dams) on the loss of freshwater biodiversity. In Australia [26], Japan [27], and the USA [28], researchers evidenced how in-stream physical barriers contribute to fish population declines or elimination. Regarding contaminant-caused fragmentation, [29] provided evidence that poor water quality in the watershed of River Scheldt in West Europe was acting as a barrier for the upstream migration of an anadromous fish. Moreover, [30] conducted laboratory avoidance tests and found that field-relevant concentrations of the herbicide atrazine might influence the spatial distribution and isolation of up and downstream fish populations. The same is true for many types of contaminants, from metals to PAH, pesticides, and even pulp mill effluents, which function as environmental stressors, causing organisms including fish and also invertebrates to prevent the exposure by mechanisms of active and passive avoidance, such as drift [31]. For example, pulses of neurotoxic insecticides have been proven to increase invertebrate downstream drift in stream mesocosm and microcosm experiments [32,33]. Drift initiated as fast as 2 h after the contamination at field-relevant concentrations, far lower than the LC50.

Therefore, the presence of a pollutant in the field might exert both a toxic effect and an avoidance-triggering effect. In this study, we hypothesized that several agriculturally related stress factors might be promoting fragmentation of the river network by posing a multi-factor pollution barrier which limits longitudinal habitat connectivity. Therefore, we aimed to identify the main factors influencing in-field ecological integrity, the fragmentation of the aquatic habitat, and the loss of MI biodiversity within a Neotropical watershed.

## 2. Materials and Methods

Study area: The Volcán River watershed is located in the south Pacific (Puntarenas province) of Costa Rica, Central America, between geographic coordinates (WGS84) −83°20.3409′ to −83°29.1345′ W; and 9°07.8204 to 9°22.3043′ N (Figure S1). It comprises a wide altitudinal range, from 221 to 3126 m.a.s.l. Consequently, this is a key watershed for connecting both terrestrial and aquatic flora and fauna between the International La Amistad Park (natural protected area), in the upper section of the basin, with coastal ecosystems in the lowlands. This watershed has highly conserved areas, mixed with pastures and coffee plantations in the upper basin and extensive pineapple and sugarcane agriculture in the middle-lower section, where the alluvial fans are formed. It extends for 22,600 ha and forms part of the Grande de Térraba River basin [34]. Mean annual precipitation in this area ranges from 3100 to 3700 mm [35]. This watershed comprises forests, small urban settlements, cattle pastures, and agriculture (mostly pineapple, sugarcane, and coffee).

Study design: This study was divided into two time periods: 1. from 2011–2013 and 2. from 2018–2019.

### 2.1. Ecological Integrity and Water Quality in the Volcán River Watershed from 2011–2013

We evaluated the ecological integrity and water quality from 2011–2013 through trimestral sampling in eight sites distributed in the Volcán (3 sites), Cañas (2), and Ángel (1) Rivers, and Peje (1) and Maura (1) streams (Figure S1). The ecological integrity was assessed by a combination of (a) habitat structure indexes (both in-stream and in the river bank), (b) biodiversity of aquatic biota (MI community sampling), and (c) anthropogenic stress (determined with basic physical and chemical parameters and pesticide residue analysis). Meanwhile, the water quality was assessed with a MI-based Biotic Index and also with the results from the physical and chemical parameters.

(a) Habitat structure: following Acosta et al. [36], two habitat indexes were used, the IHF: Fluvial Habitat Index, and the QBR-And: Riverbank Vegetation Quality Index. The IHF was estimated as a measure of in-stream habitat diversity and serves the purpose of differentiating between the effects of pollution and those of low availability of microhabitats in the rivers. The QBR-And also was estimated to state the quality of the riparian forest in the study sites.

(b) Aquatic MI community sampling and analysis: organisms were collected for 10 min using a D net (300 μm) and stored in 80% ethanol. At the Laboratory for Ecotoxicological Studies (ECOTOX) at the Universidad Nacional (UNA, Heredia, Costa Rica), the organisms were separated and identified to the lowest possible taxonomic level using a stereoscope and pertinent identification keys [37,38,39]. The abundance and richness of taxa was estimated, and the BMWP-CR biotic index [40] was calculated to determine water quality. Richness of taxa, abundance, and the BMWP-CR index were calculated also as measures of MI diversity.

(c) Physical and chemical parameters: pH, conductivity (µS/cm), temperature (°C), and dissolved oxygen (DO, mg/L) were determined in situ using a YSI 6600 portable multi-probe equipment to evaluate the basic conditions of the rivers and streams. Meanwhile, water samples were taken in 0.5 L plastic bottles and transported on ice for biological oxygen demand (BOD, mg/L), nitrate (mg/L), and total phosphorus (mg/L) analysis, to have insight into the presence of organic matter and nutrients in the water, as well as measuring anthropogenic stress. These parameters were determined at the Laboratory for Chemical Analysis and Services (LASEQ-UNA), following the Standard Methods for the Examination of Water and Wastewater [41]. For the pesticide residue analysis, surface water samples were collected by inserting pre-washed 2 L glass bottles into the water. The collected samples were transported in cooled ice boxes to the Laboratory of Pesticide Residue Analysis (LAREP-UNA) and stored at 4–6 °C for a maximum of 24 h before the analyses. In this time period (2011–2013), pesticide analyses were performed as specified in Rämö et al. [19].

### 2.2. Ecological Integrity and Water Quality in the Peje Stream from 2018–2019

We made a second sampling effort (12 monthly samples) only in one site: the Peje stream, between February 2018 to February 2019. In this opportunity, we determined the same parameters as before (IHF and QBR as habitat structure metrics; pH, conductivity, temperature, and DO as basic physical and chemical parameters; nitrates and total phosphorus as nutrient, energy, and food sources; and pesticide residue analysis to assess xenobiotic presence). However, we added new parameters such as phytoperiphyton abundance as additional energy and food sources for MI, and channel width, current velocity and flow as hydrology variables. The purpose of including these variables was to acknowledge other ecological processes as factors influencing MI community structure.

Furthermore, pesticide residue analyses were modified as follows: samples were analyzed by gas chromatography with mass detector Agilent 7890A-5975C GC-MS (Agilent Technologies, Palo Alto, CA, USA) using selective ion monitoring (SIM) and by liquid chromatography Waters Acquity UPLC H-Class with mass detector XEVO T-QS Micro, LC-MS/MS (Waters, Milford, MA, USA), using multiple reaction monitoring (MRM). The water samples, after adding internal standards, were extracted by solid-phase extraction (SPE) using previously conditioned Isolute ENV+ (200 mg/6 mL) (Biotage, Uppsala, Sweden) cartridges. For GC, the cartridge was eluted with ethyl acetate and the extract was concentrated with nitrogen and changed into isooctane, with a final volume of 0.25 mL. For LC, the same extraction procedure was followed, except that the elution was performed with methanol, and it was concentrated into methanol/water (10:90 *v*/*v* or 40:60 *v*/*v*), with a final volume of 0.5 mL. Target analytes were identified by retention times and confirmed with SIM or MRM ratios. Quantification was performed with internal and external calibration curves of the target analytes (quantification and detection limits can be found in Table S1).

Primary producer’s community sampling and analysis: phytoperiphyton was collected following the Ebro Hydrographic Confederation protocol (2005), and five rocks submerged and exposed to sunlight were collected. Using a toothbrush, a total area of 100 cm^2^ was scraped. With each scraping, the brush was placed in a bottle with 50 mL of sterile distilled water. The sample was fixed with concentrated lugol and transferred to the ECOTOX lab on ice and in darkness. With the help of a microscope, a triplicate drop of the sample was observed. Counting of cells was performed using a Neubauer counting chamber, and total abundance of phytoperiphyton was estimated.

Water flow was determined using a FH950.0 HACH digital flow meter, and the channel width, current speed (m/s), and the depth (m) of the water column were recorded in a transverse section of the channel. The distance between each measurement was 1 m. The data obtained were placed in the formula: Q = A × V, where Q represents the flow (m^3^/s), A is the area of the section of the course (mean depth times width), and V is the mean current velocity of the stream [42].

Data analysis: All measurements, determinations, and samples were collected and analyzed by Universidad Nacional laboratories with qualified personnel and methodologies. This assured uniformity of data quality irrespective of the time period of the research project.

For the pesticide residue data, measurements above the detection limits (LOD) and below the quantification limits (LOQ) were substituted with half of the LOQ, while data below the LOD (not detected) were substituted with an extremely low arbitrary value of 0.0001 µg/L.

Ordination exploratory analyses were carried out in R (R Core Team 2019) programming environment and vegan library [43,44]. Based on the biological community data matrix, we generated a detrended correspondence analysis (DCA), from which we obtained a length gradient of 3.24. Therefore, a redundancy analysis (RDA) was applied to clarify the relationships between environmental and MI community data. BOD was not used for this analysis because there was missing data in some of the sampling events from the Peje stream and RDA requires a complete dataset. The incorporation of this parameter would have implied omitting several sampling events; therefore, we decided to keep the totality of sites and sampling events, acknowledging that the exclusion of BOD might somehow affect the conclusions drawn from the RDA.

Previous to the execution of the RDA, individual pesticide concentrations were grouped and summarized according to their biocide action and their mode of action, following information from the Insecticides, Herbicides and Fungicides Resistance Action Committees (FRAC, IRAC, and HRAC) [45,46,47]. A codification was created with the initial of the biocide action: F = fungicide; H = herbicide; I = insecticide, followed by the mode of action. For example, Sum_H5 represents the addition of all concentrations of detected herbicides in a water sample, with mode of action 5 (photosystem II inhibitor; D1 Serine 264 Binders), according to HRAC [47]. Table S2 shows the represented modes of action for each pesticide active ingredient.

Before the RDA, physical and chemical variables were standardized [48] and biological data were transformed using a Hellinger transformation [49]. Variation inflation factors (VIFs) were employed to identify and eliminate variables with high collinearity [50]. To improve the model, we performed a forward selection using the adjusted R^2^ as the criteria to select the best subset of physical and chemical variables that influenced the MI data with the adespatial library [51].

For visualization purposes, the biplot cannot show all the identified taxa within the watershed; therefore, we conducted a SIMPER analysis (R^2^, *p* < 0.05) [52] to extract only the taxa that contribute >70% of the difference in the communities between dry and rainy seasons. These taxa are shown in the RDA biplot.

## 3. Results

### 3.1. Ecological Integrity and Water Quality in the Volcán River Watershed from 2011–2013

Between 2011 and 2013, 45 total samples were taken from the eight study sites in six trimestral field campaigns. The ecological quality indexes QBR-And and IHF showed their highest values in the upper basin sites, decreasing toward the lowlands. BMWP-CR index showed the highest values (good to excellent water quality) in the upper basin sites, with slightly lower values (good to regular water quality) in the Maura stream and the lower section of the Volcán River. The lowest values were calculated for the Peje stream sampling site during the rainy season (bad and very bad water quality) (Table 1).

As can be seen from Table 1, the Volcán River watershed had (in general) high ecological integrity in all sites except for the Peje stream.

Total MI identified from the Volcán River watershed accounted for *n* = 26,243 individuals, distributed in 20 orders, 75 families, and 128 genera. Number of identified families was highest in the upper basin sites of the Cañas, Ángel, and Volcán Rivers, while the lowest numbers were recorded for the Peje stream (Table 2). Only in this stream we found a marked seasonal variation in the abundance of 16 MI families (Ephemeroptera: Caenidae, Leptohyphidae, and Leptophlebiidae; Trichoptera: Glossosomatidae and Philopotamidae; Plecoptera: Perlidae; Odonata: Calopterygidae, Coenagrionidae, and Libellulidae; Coleoptera: Hydrophilidae and Staphylinidae; Megaloptera: Corydalidae; Diptera: Ceratopogonidae and Tipulidae; Lepidoptera: Crambidae; and Gastropoda: Planorbidae), whose presence was limited exclusively to the dry season (December to April), disappearing completely from the stream in the rainy season (May to November).

Overall, Cañas River sites had slightly higher pH (≈8), while Cañas and Volcán 1 sites had higher DO (≈9 mg/L), Volcán 2 and 3 had higher BOD (>5 mg/L), and the Peje stream had the highest temperature (25.4 °C), conductivity (57.83 µS/cm), and concentrations of nitrates (max 20.3 mg/L), in comparison with all the other sites within the Volcán River watershed (Table 3).

The Peje stream also had the highest concentrations of pesticide residues (mainly herbicide bromacil and organophosphate insecticide diazinon) in the first study period and throughout the complete study (Table 4 and Table S3).

Therefore, in order to better understand the ecological processes and environmental pressures taking place at the Peje stream, we made a second sampling effort with additional ecological factors determined only in this stream to complement the existing information and aid in the understanding of the seasonal absence of MI families in the rainy season.

### 3.2. Ecological Integrity and Water Quality in the Peje Stream from 2018–2019

For the period 2018–2019, the QBR-And index remained the same (45; bad riparian vegetation quality), with no detectable differences in the studied stream section. The IHF index varied from 46–64, denoting a slightly better microhabitat availability for MI in this period (Table 5). However, the BMWP-CR index was lower in 2018–2019, with a minimum score of 12 in the rainy season (extremely bad water quality) and a maximum of 76 in the dry season (regular water quality), in contrast with the maximum score of 102 (good water quality) obtained during the dry season of 2012 (see Table 1 and Table 5).

**Figure 1:**
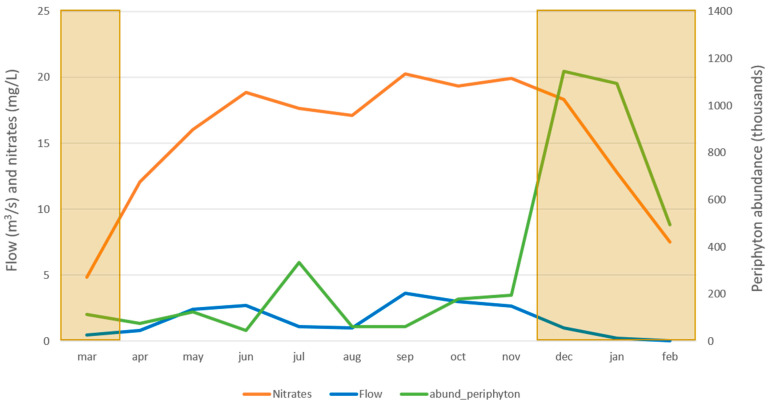
Temporal variations of the flow (m^3^/s), nitrate (mg/L), and phytoperiphyton abundance (thousands), measured in the 2018–2019 sampling period in the Peje stream. Darker boxes represent dry season periods.

Regarding physical and chemical parameters such as temperature, pH, conductivity, and DO, we determined very similar values as in 2011–2013, as well as similar values between the dry and the rainy seasons (Table 5). However, some parameters did show variation with respect to seasonality; they were the current velocity, the flow, the nitrate concentration, and the abundance of periphyton (Figure 1). A clear increment in the nitrate concentration could be seen in the rainy season, which followed the same pattern as the flow. Furthermore, phytoperiphyton abundance increased in two specific moments (July and December), when two factors happen at the same time: 1. flow starts to decrease as precipitation diminishes; and 2. there is a high concentration of nutrients (nitrates) available in the water column. The precipitation decrease in July obeys a climatic pattern called the “veranillo de San Juan”, which is a hot and dry period (usually 5–15 days long) at some point between July and August, in the middle of the rainy season.

**Figure 2:**
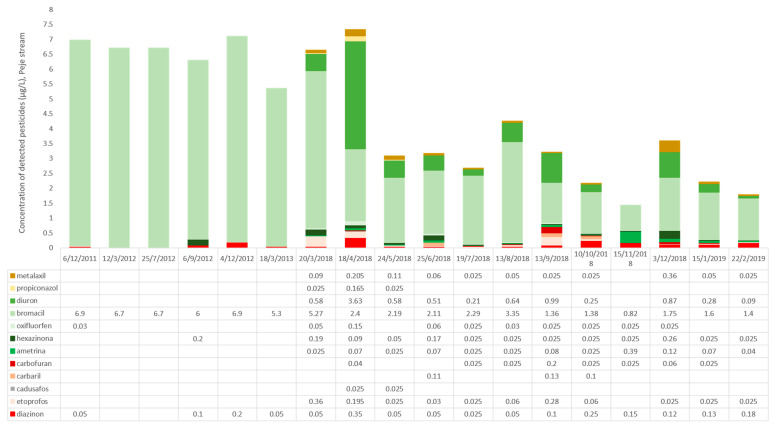
Pesticide concentrations (µg/L) detected in the Peje stream and Volcán River basin for both study periods (2011–2013 and 2018–2019). Traces (>LOD; >LOQ) were replaced by half the LOQ for each substance.

With respect to pesticide residues, fifteen pesticide active ingredients were detected in the study area, most of which are known to be applied to the major crops in this watershed (pineapple and sugarcane). From these pesticides, cadusafos and carbofuran were only analyzed in the 2018–2019 period, when a change in the methodology allowed the determination of more substances and at lower concentrations. Therefore, we cannot discuss or compare their detection between both study periods. However, similar to the 2011–2013 sampling period, herbicides had the highest concentrations, followed by insecticides. The major difference between these periods was the decrease in the concentration of bromacil and the increment in the concentrations of insecticides highly toxic for aquatic organisms (carbaryl, ethoprophos, and diazinon). Additionally noteworthy is that several new substances (including fungicides) were detected only in the second study period (Figure 2 and Table S3). Nevertheless, we did not observe a clear trend of pesticide concentrations increasing or decreasing according to the precipitation regimes or seasonality. Pesticides in the Peje stream were present throughout the year in similar concentrations in both study periods (Figure 2). 

### 3.3. Relationships between Environmental Variables and Macroinvertebrate Community Data

**Figure 3:**
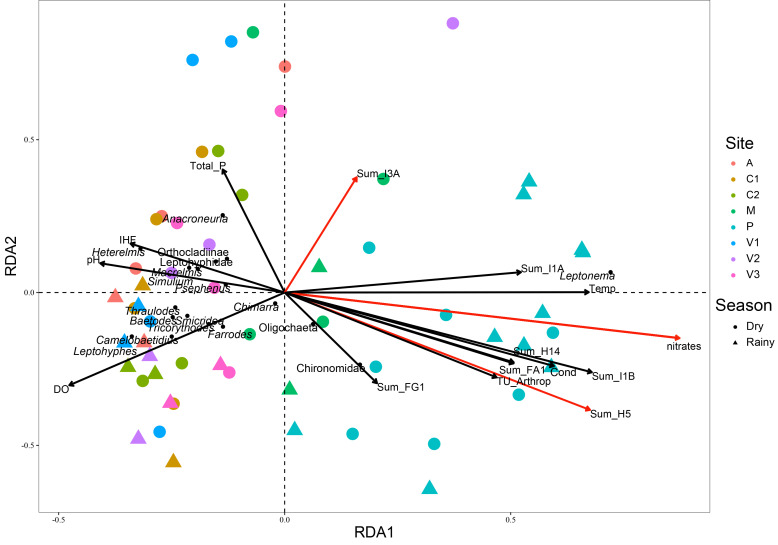
RDA biplot showing the relationships between environmental and macroinvertebrate community data in the Volcán River watershed. The ordination of sites with respect to the explanatory variables and selected taxa are shown. Labels: A = Angel, C = Cañas, M = Maura, P = Peje, and V = Volcán. Marked in red are the most relevant explanatory variables.

We aimed to better analyze the complete dataset (study period 1 and 2) with the help of an RDA, as detailed in the methodology. This RDA model (F = 3.17, gl. = 13, 43; *p* = 0.001) and both axes (RDA 1: F 20.89; *p* = 0.01; RDA 2: F = 5.03, *p* = 0.01) explained 33% of the variation in the MI communities (adjusted R^2^ = 0.33). According to the forward selection method, we selected the best subset of physical and chemical variables that influenced the composition of the MI community in the Volcán River watershed, and they were: 1. nitrates, 2. Sum_H5 (herbicides ametryn, bromacil, diuron, hexazinone, and terbutryn), and 3. Sum_I3A (permethrin) (Figure 3). As can be seen in the biplot, the Peje stream MI community was separated from all the other sites in the watershed. At the same time, the differences between the dry and rainy season are also reflected in the RDA biplot. MI taxa in Figure 3 are the ones which contributed to 70% of the difference between dry and rainy seasons. Only a few taxa such as *Leptonema* (Trichoptera: Hydropsychidae) or Chironomidae (Diptera) were found all year round in the Peje stream during the sampling periods (Table S4). This biplot also highlights the increased number of stressors affecting the MI of the Peje stream in comparison with all the other sites in the watershed.

## 4. Discussion

As we saw from this study, agricultural pollutants (mainly nitrates, herbicides, and insecticides) have produced a fragmentation of the continuum (capacity of maintaining lateral and longitudinal connectivity for biota) of the Peje stream river network, and this has negatively affected its biodiversity.

In river networks, barriers exist of different types (natural and anthropogenic) and degrees of permeability (how much they block movement of organisms), and have divided the habitat into very small patches [53] which are less resilient, as they interact with other stressors. In our study, the Volcán River watershed had large areas of forest, excellent water quality, and riparian vegetation in most of its rivers, which could function as refuge areas [54] that allow for the re-colonization of organisms into the Peje stream. However, those organisms were incapable of continuous development and growth, providing evidence that the movement of organisms upstream from the main Volcán River into Peje stream affluent is impeded by a chemical habitat barrier that prevents life of the most sensitive organisms, even when the structural habitat conditions might be good and diverse. As stated by Araújo et al. [31], contaminants act as habitat disturbers or fragmentors, by promoting active and passive avoidance responses that end up generating uninhabited zones due to local population extinctions.

According to our results, the nitrate concentration in the Peje stream was the major disruptor for connectivity during the rainy seasons of both study periods. Nitrates followed the same pattern as the flow, which is an indication that the main source of this nitrogen is runoff from the crop fields, due to the extensive use of fertilizers and their high solubility in water, a problem well-documented worldwide since decades ago [55,56,57,58] and still relevant [59].

With increasing precipitation and runoff, an increment in the flow, the load of suspended solids, and the turbidity is also expected in the water courses [58]. Such a situation decreases the penetration of light through the water column, altering photosynthesis and lowering primary production [60], which is what we see happening in the Peje stream during the rainy season, when the lowest abundance of phytoperiphyton is registered in accordance with the higher peaks in flow.

On the contrary, the highest abundances of phytoperiphyton are registered when precipitation diminishes, with the consequent decrease in flow, and when sunlight can penetrate further into a nutrient-filled water column (highest concentrations of nitrates). Such nutrients cannot be used by the primary producers when the light penetration is low, but are rapidly consumed as soon as the flow and turbidity decrease in the stream and the higher photosynthesis rates accelerate the reproduction of periphyton [61]. These primary producers might be helping to increase MI taxa richness and abundance in two ways: 1. by uptake of the excess nitrogen from the water column, and 2. serving as food source for any re-colonizing organisms.

It is noteworthy to mention that the herbicide bromacil, which was the most detected pesticide in the first study period and was normally used in pineapple crops in the past, was forbidden in Costa Rica in 2017 [62], and this circumstance explains both the decreased detections in the 2018–2019 period and the increased appearance of other herbicides with the same mode of action, such as diuron, ametryn, and oxyfluorfen. The effect exerted by the constant presence of those herbicides in high concentrations on the diversity and abundance of the primary producers was not clear in this research. However, some studies [63] have indicated the possibility that toxic effects of herbicides on primary producers are obscured by the over-abundance of otherwise limiting nutrients (such as nitrates or phosphorus); or by the bioavailability, uptake, and toxicity of herbicides and their metabolites, which depend on factors such as temperature, pH, and DO concentrations; or due to pollution-induced community tolerance [64]. Therefore, it remains a challenge to understand the dynamics between energy sources and herbicide presence in the aquatic ecosystems overall, their direct effects on primary producers, and the indirect effects in upper trophic levels, particularly in the tropical areas.

Even though nitrates were evidenced in this study as the major pollutant affecting ecological integrity and biodiversity in the Peje stream during the rainy season, the RDA reflects that pesticide presence is certainly an aspect to continue evaluating. Although nitrate concentration ranges did not change between both sampling periods, the MI community of the Peje stream was even less diverse in 2018–2019 than in 2011–2013, as mirrored by lower BMWP-CR index scores. Moreover, MI families such as Perlidae (Plecoptera), Psychodidae (Diptera), Ptilodactylidae (Coleoptera), Gomphidae (Odonata), Leptoceridae, and Glossosomatidae (Trichoptera), which were collected in the first study period, were no longer present in the second. Some of these orders have been identified as sensitive to pesticides [65,66,67] or have been negatively correlated with pesticide exposure in the Caribbean region of Costa Rica [16], and their absence might be related to the presence of higher concentrations of toxic organophosphate and carbamate insecticides. On the contrary, the families inhabiting the Peje stream all year round (mainly Hydropsychidae and Chironomidae, but also Elmidae, Gerridae, Hydroptilidae, and Simuliidae) can be considered tolerant to the prevailing conditions (elevated nitrate and herbicide or insecticide concentrations). The Species at Risk (SPEARpesticide) index [65,66] identifies taxa that are at a higher risk of being affected by pesticide pollution. This approach classifies Hydropsychidae, Chironomidae, and Elmidae as species not at risk, in accordance with the present study, while Hydroptilidae is identified as a taxon at risk, contrary to our findings.

There is a gap in knowledge on the sensitivity of tropical MI toward pesticides, which needs to be filled in order to better understand the risks of these substances in conjunction with accompanying stressors. Another study by Alexander et al. [68] also found a MI community level response driven by the combined effect of nutrients and the insecticide imidacloprid in experimental outdoor artificial streams.

In the Volcán watershed, the maximum concentration of several of the detected pesticides (diazinon, ethoprophos, cadusafos, chlorpyrifos, permethrin, ametryn, bromacil, and diuron) surpassed international environmental quality standards (EQS; see Table S3) [69,70] and represents a risk for the aquatic ecosystem. Moreover, the concentrations necessary to produce an avoidance effect are far lower than the ones needed to produce toxicity [30,31,32,33]. It would be important to further understand the most relevant pathways of the used pesticides from the crop fields into the watercourses, and how this process can be reduced as a mitigation strategy [71]. For example, Bereswill et al. [7] evidenced that drainage systems rapidly transport nutrients and xenobiotics to surface waters, lowering the natural retention capacity of catchments and the efficiency of riparian forests as buffer strips.

In a recent review, Carstensen et al. [57] reported very positive evidence that diffusive nutrient losses from agricultural systems can be mitigated by >40% with different denitrification treatment measures (free water surface constructed wetlands, controlled drainage, and buffer zones). Such denitrification is highly controlled by temperature, with higher rates in high temperature conditions, which can be an advantage if a treatment is put into place in tropical ecosystems. They also stated that these measures can provide other ecosystem services such as storage of water or even biomass production.

Alternative to the construction of denitrification systems, the reduction in applied fertilizers in the crop fields, as well as restoration of previously existing lagoons and the riparian habitats alongside the watercourses, may serve the purpose of buffer zones, temperature control, sediment and nutrient retention, and food source and habitat diversification for the biota [4,56,72,73]. Therefore, the protection of the riparian vegetation may sensibly improve the habitat conditions for all aquatic organisms, and at the same time diminish the effects of agricultural activities, as has been confirmed by [74] for Brazilian and Paraguayan streams. This measure also favors connectivity by means of riverine biological corridors.

Another relevant aspect to this area is that Central America has been identified as one of the regions with the largest climate change impact, with either precipitation reductions or increments of up to 20%, depending upon the specific geographic area [75]. The south Pacific of Costa Rica (where the Volcán River watershed is located) is predicted to have high variability and increased precipitation [76]; therefore, mitigation strategies are particularly relevant given that stream impairment and habitat fragmentation due to high concentrations of nitrates (and pesticide residues) in surface waters are related to increased runoff and flow during the rainy season.

This investigation can be used as a baseline of information for follow-up monitoring and evaluation of restoration goals. We also encourage the implementation of passive alternatives and wonder: is it possible to see the recovery of the Peje stream ecosystem after only agricultural abandonment of key zones within this sub-watershed? This type of follow-up study is considered a major gap in our current understanding of stream management [21].

## 5. Conclusions

In this study, we provided evidence that agriculturally related contaminants might drive fragmentation of the habitat and can produce MI biodiversity loss in the field. However, fragmentation has been known and studied almost exclusively for terrestrial ecosystems, contributing to an underestimation of the threats posed to aquatic biota [25]. Up until this date, the vast majority of the research on river network fragmentation worldwide has been focused on the effects of barriers on fish populations; however, we believe that research should advance toward the understanding of the effects on other types of organisms, as well as making the evaluations at the watershed level, rather than studying only individual barriers.

Agricultural contaminants (in this case, the concentration of nitrates and pesticides) are causing an abrupt rupture of the ecological integrity of a stream and a seasonal loss of MI biodiversity. The large effect observed for nitrates might even obscure the effects produced by other highly relevant stressors in the aquatic ecosystems, such as pesticides or even modifications in the river channel morphology. Therefore, we encourage more researchers to incorporate the evaluation of the effects of fertilizer runoff on higher trophic levels and not only in primary producers, and also to provide that information to improve regulatory guidelines. The EU Nitrates Directive [59] states that this is a major water pollution problem in Europe and represents an obstacle to reach “good status” for all surface waters. In our study, the maximum nitrate concentration was 20.3 mg/L, less than half of the 50 mg/L needed to be considered a Nitrate Vulnerable Zone within this directive [59].

Chemical habitat fragmentation might be relevant as a biodiversity loss factor in many watersheds in the world, which may only have been analyzed from a water quality or ecotoxicological point of view, with disregard of the effects that the fragmentation *per se* can pose on the aquatic populations in the long term. It is necessary to conduct research leading to design and validation of stream restoration strategies based on field data (social and ecological inputs) with a river ecology focus, directed toward barrier removal to re-establishing aquatic species diversity and ecosystem functioning.

Finally, as attested by Fuller et al. [25], watershed management with biodiversity conservation goals requires the acknowledgement of the aquatic habitat fragmentation concept in order to avoid underestimation of its effects and to take specific fragmentation management actions.

## Figures and Tables

**Table 1:** Ecological quality index values (QBR-And, IHF (min–max), and BMWP-CR) calculated in 8 sites from the Volcán River watershed, period 2011–2013.

Basin Position	QBR-And	IHF	Site	Dec-11	Mar-12	Jul-12	Sep-12	Dec-12	Mar-13
Upper	95	59–67	Volcán 1	174	158	118	129	139	108
Upper	75	53–66	Angel	-	125	122	158	140	106
Upper	95	58–74	Cañas 1	153	164	160	195	164	132
Middle	75	47–56	Volcán 2	141	111	131	142	157	-
Middle	90	36–48	Maura	106	113	102	117	83	87
Middle	45	41–47	Peje	30	102	47	19	-	91
Lower	85	48–56	Volcán 3	136	140	140	112	120	89
Lower	70	51–73	Cañas 2	140	187	123	126	122	122

QBR color interpretation: green = good vegetation quality; yellow = intermediate vegetation quality; orange = bad vegetation quality [36]. IHF < 40 = inadequate to support a diverse MI community [36]. BMWP-CR color interpretation: dark blue: excellent water quality; light blue: good water quality; green: regular water quality; yellow: bad water quality; orange: very bad water quality [40].

**Table 2:** Summary of benthic macroinvertebrate families and seasonality pattern in the sampling sites, period 2011–2013. Number of total identified macroinvertebrate families per site and seasonality pattern of registered families.

Basin Position	Site	Total Identified Families	Present in >50% of Samples	Present Only in the Dry Season	% of Families Showing Seasonality
Upper	Volcán 1	46	25	2	4
Upper	Angel	49	22	7	14
Upper	Cañas 1	50	31	3	6
Middle	Volcán 2	35	20	0	0
Middle	Maura	40	18	5	13
Middle	Peje	32	9	16	53
Lower	Volcán 3	48	23	5	10
Lower	Cañas 2	51	26	6	12

**Table 3:** Mean and standard deviation (mean ± standard deviation) of physical, chemical, and nutrient parameters in the Volcán River watershed, period 2011–2013.

Site	Temp (°C)	pH	Cond (µS/cm)	DO (mg/L)	BOD (mg/L)	Nitrates (NO_3_; mg/L)	Total P (mg/L)
Volcán 1	20.1 ± 1.3	7.3 ± 0.3	46.0 ± 5.1	8.4 ± 0.4	3.93 ± 2.15	0.38 ± 0.31	3.79 ± 8.39
Angel	22.0 ± 1.6	7.0 ± 0.3	26.8 ± 5.0	8.2 ± 0.4	3.33 ± 1.27	0.38 ± 0.31	0.03 ± 0.03
Cañas 1	19.6 ± 1.2	7.5 ± 0.4	35.8 ± 3.1	8. 5 ± 0.3	3.2 ± 2.36	0.38 ± 0.31	1.59 ± 3.47
Volcán 2	24.3 ± 2.3	7.3 ± 0.3	37.6 ± 4.6	8.2 ± 0.2	5.5 ± 4.86	0.47 ± 0.33	1.39 ± 2.74
Maura	24.5 ± 0.7	6.7 ± 0.4	22.1 ± 6.3	7.7 ± 0.3	3.4 ± 2.77	1.64 ± 0.93	0.44 ± 0.87
Peje	25.4 ± 1.2	7.0 ± 0.8	57.8 ± 7.3	8.0 ± 0.3	4.2 ± 2.28	13.78 ± 6.08	0.1 ± 0.2
Volcán 3	24. 6 ± 2.0	7.3 ± 0.5	38.8 ± 4.7	8.2 ± 0.3	5.73 ± 5.1	3.71 ± 5.26	0.77 ± 1.64
Cañas 2	22.7 ± 1.5	7.4 ± 0.3	36.1 ± 2.7	8.4 ± 0.3	3.13 ± 2.01	1.07 ± 1.66	0.04 ± 0.04

**Table 4:** Pesticide residues detected in the Volcán River watershed (2011–2013). Concentrations (µg/L) are presented as min–max (no. detections). Where no interval and parenthesis are presented, only one detection was made.

Site	Diazinon	Terbutryn	Bromacil	Oxyfluorfen	Hexazinone	Permethrin
Volcán 1	nd	nd	nd	nd	nd	nd
Angel	nd	T	nd	nd	nd	0.4
Cañas 1	nd	T	nd	nd	0.3	nd
Volcán 2	T	nd	0.1–0.14 (3)	nd	nd	T
Maura	T	T	0.21–1.2 (4)	nd	nd	nd
Peje	0.05–0.2 (4)	nd	5.3–6.9 (6)	T	0.2	nd
Volcan 3	T–0.02 (4)	T	0.6–1.3 (5)	nd	nd	nd
Cañas 2	T	nd	nd	nd	nd	nd

nd = below detection limit. T = between LOD and LOQ.

**Table 5:** Minimum, maximum, mean, and standard deviation of the variables measured or estimated in the 2018–2019 sampling period in the Peje stream. Information is presented separately for the dry and rainy seasons.

Parameter	Dry Season				Rainy Season			
	MIN	MAX	MEAN	SD	MIN	MAX	MEAN	SD
IHF score	50	64	58.40	5.13	46	62	52.57	6.35
BMWP-CR	21	76	51.8	26.44	12	62	24.71	17.38
Taxa richness MI	4	24	15.4	9.6	3	25	8.4	7.6
Temperature (°C)	23.1	26.3	24.88	1.25	24.2	26.5	25.19	0.81
pH	6.68	8.84	7.32	0.89	5.7	7.22	6.58	0.58
Conductivity (µS/cm)	55.5	73.4	63.98	7.37	47.3	58.7	52.09	3.52
DO (mg/L)	7.72	8.66	7.99	0.39	7.54	8.5	7.99	0.31
Nitrates (mg/L)	4.85	19.92 *	12.68	6.56	12.08	20.26	17.33	2.72
Total P (mg/L)	0.0201	0.075	0.05	0.039	0.052	0.115	0.074	0.036
Channel width (m)	8.2	11.5	10.06	1.47	7.1	11	8.86	1.28
Velocity (m/s)	0.03	0.43	0.24	0.18	0.39	0.78	0.55	0.15
Flow (m^3^/s)	0.04	2.66	0.88	1.06	0.81	3.65	2.11	1.12
Periphyton abundance	114 × 10^3^	1144 × 10^3^	608 × 10^3^	487 × 10^3^	45.5 × 10^3^	333 × 10^3^	126 × 10^3^	103 × 10^3^

* Concentration of the first month of the transition between rainy and dry seasons.

## Data Availability

The data presented in this study are available on request from the corresponding author.

## Supplementary Materials

The following supporting information can be downloaded at: https://www.mdpi.com/article/10.3390/toxics10070346/s1, Figure S1: Map of the Volcán River watershed and sampling sites, Table S1: Pesticide residue limits of detection (LOD) and of quantification (LOQ) in both study periods (2011–2013 and 2018–2019) in µg/L. Active ingredients are ordered alphabetically, Table S2: Pesticide active ingredient mode of action according to Fungicides, Herbicides and Insecticides Resistance Action Committees FRAC/HRAC/IRAC, Table S3: Maximum detected concentration (µg/L) of pesticide active ingredients in the Volcán River watershed, 2011–2013 (all sites) and 2018–2019 (only Peje stream). nd = below detection limit. na = not analyzed, Table S4: Macroinvertebrate families identified in each sampling campaign in the Peje stream (2011–2013 and 2018–2019). Yellow = dry season; green = transition; blue = rainy season.
