# Supplementary material for: Ecological Integrity Impairment and Habitat Fragmentation for Neotropical Macroinvertebrate Communities in an Agricultural Stream
Source: Toxics. 2022 Jun 22;10(7):346. doi: 10.3390/toxics10070346 (PMC9316105; doi:10.3390/toxics10070346)
Supplement: Supplementary file 1 [file toxics-10-00346-s001.zip › toxics-1761465-supplementary.pdf]

toxics

MDPI

Supplementary Materials: Ecological Integrity Impairment and Habitat Fragmentation for Neotropical Macroinvertebrate Communities in an Agricultural Stream

Silvia Echeverría-Sáenz, Rocío Ugalde-Salazar, Meyer Guevara-Mora, Francisco Quesada-Alvarado and Clemens Ruepert

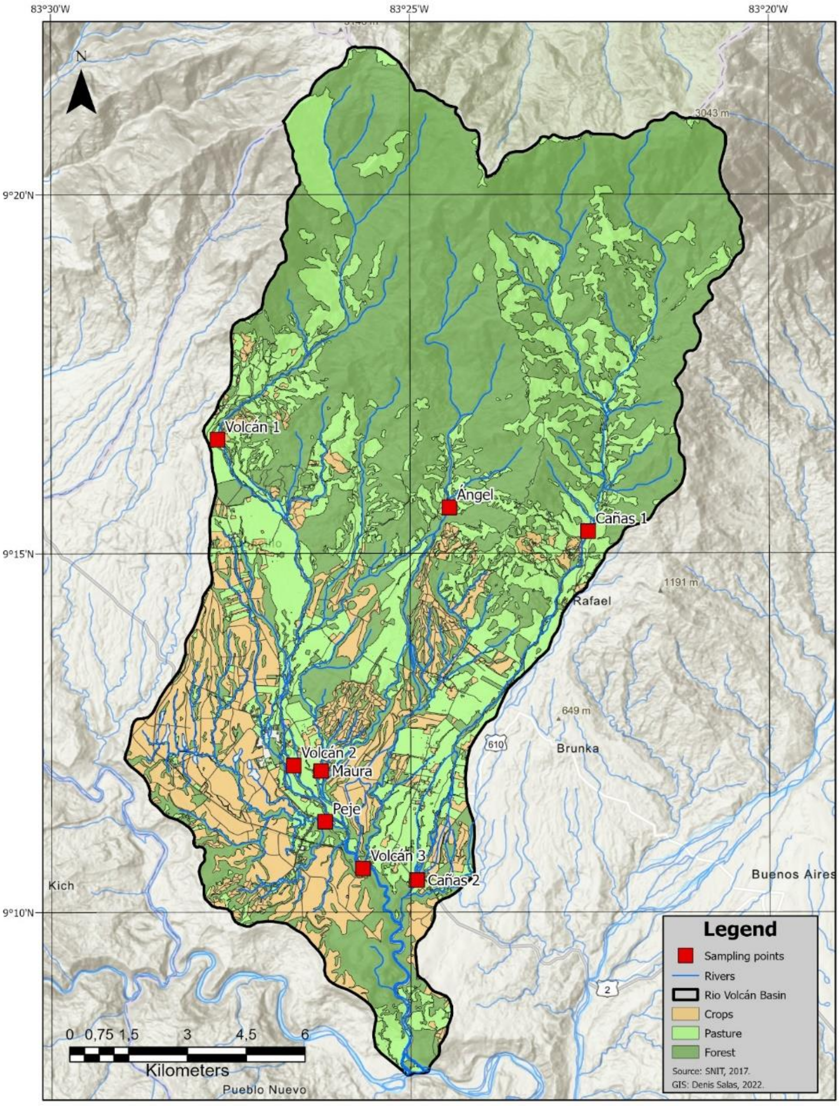

Figure S1. Map of the Volcán River watershed (South Pacific, Costa Rica) and sampling sites.

Table S1. Pesticide residue limits of detection (LOD) and of quantification (LOQ) in both study periods (2011-2013 and 2018-2019) in μg/L. Active ingredients are ordered alphabetically.

Active ingredient.	Volcán (2011-2013)		Q_Peje (2018-2019)	
	LOD	LOQ	LOD	LOQ
a-cypermethrin	0.03	0.1	0.02	0.05
ametryn	0.02	0.05	0.01	0.03

**Active ingredient.**	**Volcán (2011-2013)**		**Q_Peje (2018-2019)**	
	**LOD**	**LOQ**	**LOD**	**LOQ**
atrazine	0.02	0.05	0.02	0.05
azoxystrobin			0.02	0.05
bentazone			0.02	0.05
bifenthrin	0.1	0.5	0.02	0.05
bitertanol	0.06	0.2	0.02	0.05
boscalid			0.02	0.05
bromacil	0.03	0.1	0.02	0.05
buprofezin			0.02	0.05
butachlor	0.2	0.6	0.02	0.05
cadusafos			0.02	0.05
carbaryl	0.06	0.2	0.02	0.05
carbendazim			0.02	0.05
carbofuran			0.02	0.05
cyhalothrin	0.04	0.1	0.02	0.05
cyproconazole			0.02	0.05
clomazone			0.02	0.05
chlorothalonil	0.03	0.1	0.01	0.02
chlorpyrifos	0.02	0.05	0.01	0.02
DDD-pp(M)			0.02	0.05
DDE-pp(M)			0.02	0.05
deltamethrin	0.06	0.2	0.02	0.05
diazinon	0.01	0.02	0.01	0.03
difenoconazole			0.02	0.05
dimethoate	0.04	0.1	0.02	0.05
diuron	0.03	0.1	0.02	0.05
endosulfan-a	0.04	0.1	0.02	0.05
endosulfan-b	0.04	0.1	0.02	0.05
endosulfan sulfate (M)			0.02	0.05
epoxiconazole	0.06	0.2	0.02	0.05
ethoprophos	0.04	0.1	0.01	0.04
fenamiphos	0.3	1	0.02	0.05
fenbuconazole			0.02	0.05
fenpropimorph			0.02	0.05
fenthion	0.04	0.1	0.02	0.05
fluopyram			0.02	0.05
flutolanil			0.02	0.05
phorate	0.04	0.1	0.02	0.05
hexachlorobenzene			0.02	0.05
hexazinone	0.05	0.1	0.02	0.05
imidacloprid			0.02	0.05
lindane			0.02	0.05
linuron			0.02	0.05
malathion	0.03	0.1	0.02	0.05
metalaxyl	0.1	0.2	0.02	0.05
myclobutanil			0.02	0.05
oxifluorfen	0.06	0.2	0.02	0.05
parathion-methyl	0.06	0.2	0.02	0.05
pendimethalin	0.03	0.1	0.02	0.05
pentachloroaniline (M)			0.02	0.05
pentachloroanisol (M)			0.02	0.05
entachlorobenzene (M)			0.02	0.05
permethrin	0.03	0.1	0.02	0.05
pyrimethanil			0.02	0.05
prochloraz	0.3	1		
propanil	0.2	0.6	0.03	0.1
propiconazole	0.05	0.2	0.02	0.05

Active ingredient.	Volcán (2011-2013)		Q_Peje (2018-2019)	
	LOD	LOQ	LOD	LOQ
quintozene (PCNB)			0.02	0.05
spiroxamine			0.02	0.05
tebuconazole	0.05	0.2	0.02	0.05
terbufos	0.03	0.1	0.02	0.05
terbuthylazine	0.03	0.05	0.02	0.05
terbutryn	0.03	0.1	0.02	0.05
thiabendazole			0.02	0.05
triadimefon	0.3	1	0.02	0.05
triadimenol	0.3	1	0.02	0.05
triazophos			0.02	0.05
trifloxystrobin			0.02	0.05

Table S2. Pesticide active ingredient Mode of Action according to Fungicides, Herbicides and Insecticides Resistance Action Committees FRAC/ IRAC/ HRAC [45,46,47].

Active ingredient	CAS number	Biocide action	Chemical group	FRAC/HRAC/ IRAC MoA code	MoA Description
metalaxyl	57837-19-1	fungicide	acylalanine	FA1	protein synthesis inhibitor
propiconazole	60207-90-1	fungicide	triazole	FG1	demethylation in sterol biosynthesis inhibitor
oxyfluorfen	42874-03-3	herbicide	diphenilether, chlorinated, fluorated	H14	Protoporphyrinogen Oxidase inhibitor
ametryn	834-12-8	herbicide	triazine	H5	photosystem II inhibitor (D1 Serine 264 Binders)
bromacil	314-40-9	herbicide	uracil, bromated	H5	photosystem II inhibitor (D1 Serine 264 Binders)
diuron	330-54-1	herbicide	urea, chlorinated	H5	photosystem II inhibitor (D1 Serine 264 Binders)
hexazinone	51235-04-2	herbicide	triazinone	H5	photosystem II inhibitor (D1 Serine 264 Binders)
terbutryn	886-50-0	herbicide	triazine	H5	photosystem II inhibitor (D1 Serine 264 Binders)
carbaryl	63-25-2	insecticide	carbamate	I1A	Acetylcholinesterase inhibitor
permethrin	52645-53-1	insecticide	pyrethroid, chlorinated	I3A	Sodium channel modulator (blocks nervous stimuli)
carbofuran	1563-66-2	insecticide, nematicide	carbamate	I1A	Acetylcholinesterase inhibitor
cadusafos	95465-99-9	insecticide, nematicide	organophosphate	I1B	Acetylcholinesterase inhibitor
diazinon	333-41-5	insecticide, nematicide	organophosphate	I1B	Acetylcholinesterase inhibitor
ethoprophos	13194-48-4	insecticide, nematicide	organophosphate	I1B	Acetylcholinesterase inhibitor

Table S3. Maximum detected concentration and EQS* (µg/L) of pesticide active ingredients in the Volcán River watershed, 2011-2013 (all sites) and 2018-2019 (only Peje stream). nd = below detection limit. na = not analyzed.

Active ingredient	Volcán 1	Volcán 2	Volcan 3	Angel	Cañas 1	Cañas 2	Maura	Peje	EQS*
diazinon	nd	0.01	0.15	nd	nd	0.01	0.01	0.35	0.037
ethoprophos	nd	nd	nd	nd	nd	nd	nd	0.36	0.063
cadusafos	na	nd	nd	na	na	na	na	0.025	0.023

carbaryl	na	nd	0.025	na	na	na	na	0.13	0.23
carbofuran	nd	nd	0.025	nd	nd	nd	nd	0.2	0.91
permethrin	nd	0.02	nd	0.4	nd	nd	nd	nd	0.0003
chlorpyrifos	nd	nd	0.08	nd	nd	nd	nd	nd	0.03
ametryn	nd	nd	nd	nd	nd	nd	nd	0.39	0.01
hexazinone	nd	nd	0.025	nd	0.3	nd	nd	0.26	0.56
oxifluorfen	nd	nd	nd	nd	nd	nd	nd	0.15	-
bromacil	nd	0.14	1.3	nd	nd	nd	1.2	6.9	0.0068
diuron	nd	nd	0.35	nd	nd	nd	nd	3.63	0.2
terbutryn	nd	nd	0.05	0.05	0.05	nd	0.05	nd	0.065
propiconazole	nd	nd	nd	nd	nd	nd	nd	0.165	10
metalaxil	nd	nd	0.025	nd	nd	nd	nd	0.36	46

* EQS refers to AA or MAC Environmental Quality Standards of the European Union (or the MTR eco, when the EQS is not available) [69].

Table S4. Macroinvertebrate families identified in each sampling campaign in the Peje stream (2011-2013 & 2018-2019). Yellow =dry season; Green= Transition; Blue = rainy season.

2011-2013	dec-2011	mar-12	jul-12	sep-12	mar-13
Ampullaridae					X
Baetidae		X	X		X
Bulinidae		X			
Caenidae		X			
Calopterygidae					X
Ceratopogonidae		X			X
Chironomidae	X	X	X	X	X
Coenagrionidae		X			X
Corydalidae	X				X
Crambidae		X			
Elmidae	X	X	X	X	X
Empididae	X	X	X		X
Glossossomatidae		X			
Gomphidae			X		
Hydropsychidae	X	X	X	X	X
Hydroptilidae		X		X	X
Leptoceridae			X		
Leptohyphidae		X			X
Leptophlebiidae		X			X
Libellulidae		X			
Oligochaeta	X	X		X	
Perlidae					X
Philopotamidae		X			X
Psychodidae		X	X		X
Ptilodactylidae	X				
Simuliidae		X	X		X
Staphylinidae		X			
Tipulidae		X			
Trombidiformes			X		X
Turbellaria		X			
Veliidae		X			X

Note: No sample is available for December 2012.

Table S4 Cont.: Macroinvertebrate families identified in each sampling campaign in the Peje stream (2011–2013 and 2018–2019). Yellow = dry season; green = transition; blue = rainy season.

2018-2019	19_mar_2018	18_apr_2018	22_may_2018	26_jun_2018	17_jul_2018	13_aug_2018	12_sep_2018	10_oct_2018	14_nov_2018	03_dec_2018	14_jan_2019	21_feb_2019
adult_Diptera	X											
Baetidae	X			X	X						X	X

**2018-2019**	**19_mar_2018**	**18_apr_2018**	**22_may_2018**	**26_jun_2018**	**17_jul_2018**	**13_aug_2018**	**12_sep_2018**	**10_oct_2018**	**14_nov_2018**	**03_dec_2018**	**14_jan_2019**	**21_feb_2019**
Caenidae	X	X									X	
Calopterygidae		X									X	X
Ceratopogonidae											X	
Chironomidae	X	X	X		X	X	X	X		X	X	X
Coenagrionidae	X								X			X
Corydalidae	X			X								
Crambidae					X		X			X		
Culicidae											X	
Curculionidae										X		
Dryopidae											X	
Elmidae	X	X	X		X	X					X	
Empididae										X	X	
Gerridae	X	X	X		X	X					X	X
Hydropsychidae	X	X	X	X	X	X	X	X	X	X	X	X
Hydroptilidae	X	X				X		X	X	X		X
Hydroscaphidae												X
Leptohyphidae	X	X									X	X
Leptophlebiidae	X	X									X	X
Libellulidae	X	X		X					X		X	X
Ochteridae	X											
Oligochaeta	X	X										X
Ostracoda	X	X										
Philopotamidae		X									X	X
Platystictidae												X
pupae_Diptera	X	X	X			X					X	X
Simuliidae		X	X	X	X						X	X
Staphylinidae												X
Tabanidae											X	X
Tipulidae												X
adult_Trichoptera						X						
Veliidae	X	X									X	X

Note: Lower precipitations occur in July, because of a weather condition called the "veranillo".
